# Supplementary material for: Influence of the Peripheral Nervous System on Murine Osteoporotic Fracture Healing and Fracture-Induced Hyperalgesia
Source: Int J Mol Sci. 2022 Dec 28;24(1):510. doi: 10.3390/ijms24010510 (PMC9820334; doi:10.3390/ijms24010510)
Supplement: Supplementary file 1 [file ijms-24-00510-s001.zip › ijms-2072254-supplementary.pdf]

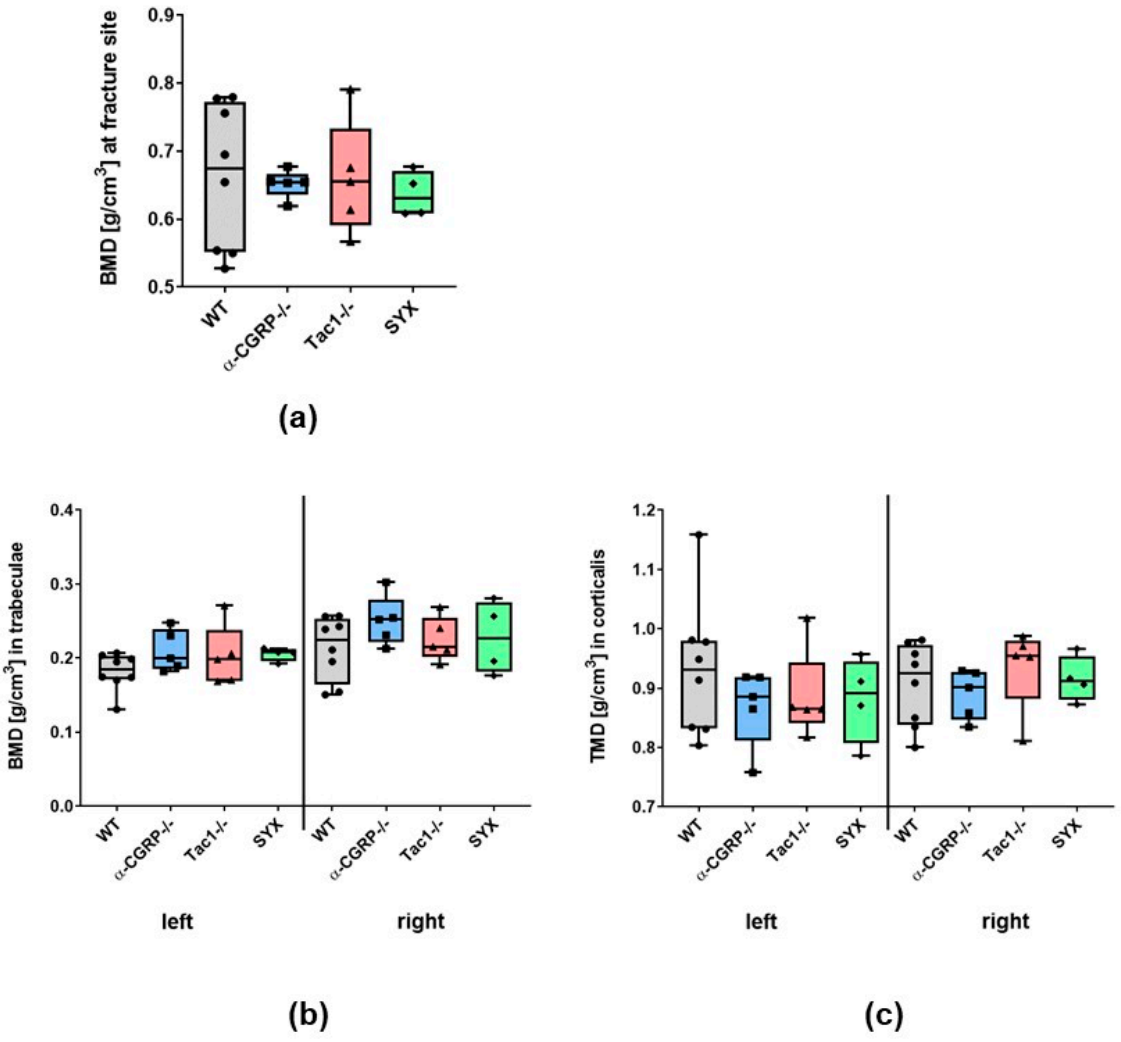

Figure S1: Measurement of bone mineral density (BMD; g/cm^3; (a), (b)) and tissue mineral density (TMD; g/cm^3; (c)) at the fracture site ((a), (b) left, (c) left) and in the contralateral, non-fractured femora ((b) right, (c) right) of WT, α-CGRP-/-, Tac1-/-, and SYX mice at 21 days after fracture. (a)BMD of the trabeculae at fracture site (left femora) between the cortical bone ends. (b) BMD in the trabecular bone of the distal fractured (left) and contralateral, non-fractured (right) femora. (c) TMD in the cortical bone of the fractured (left) and contralateral, non-fractured (right) femora. (n_{WT}=8, $n_{CGRP-/-}$=5, $n_{Tac1-/-}$=5, n_{SYX}=4).

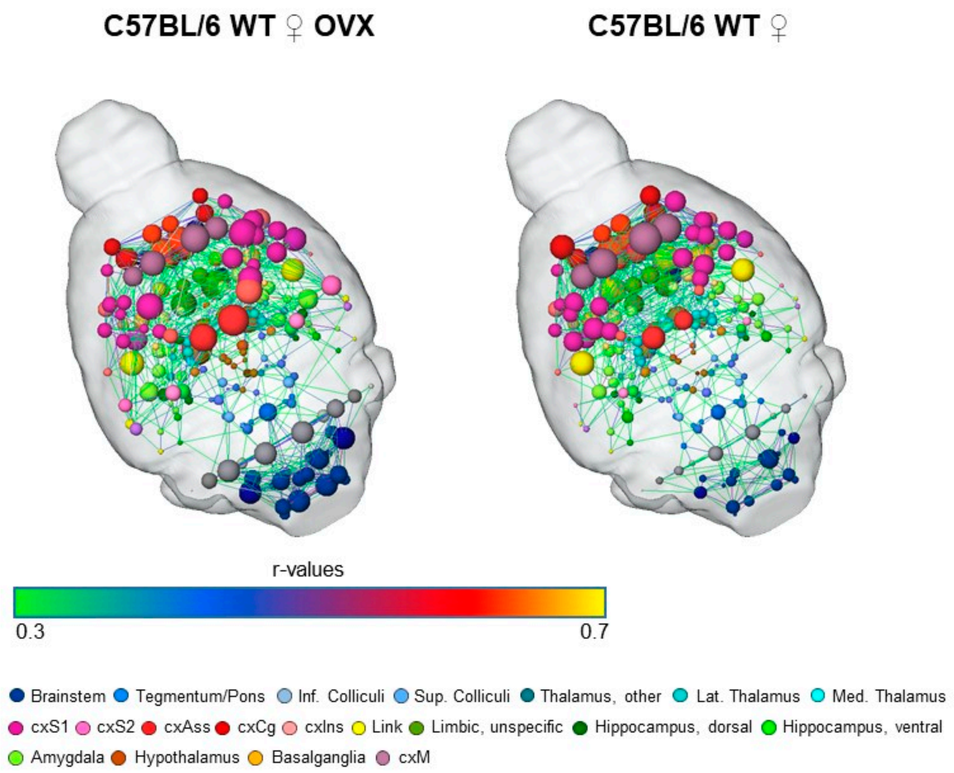

Figure S2: Resting-state (RS) functional connectivity brain networks of female C57BL/6 WT mice w/o OVX.

Shown are resting-state brain networks of the ovariectomized wild type group at day -2 before fracture (left) and comparable female mice with C57BL/6 background of a different study (right). Networks show the average Pearson r-value for each connection (edges). Networks were limited to a density of 5%, and only connections with r-values higher than 0.3 are displayed, as native RS networks are very dense.

Brain regions are displayed as color-coded nodes. The size of the node represents the degree, i.e., how many connections this node has to others. Unconnected nodes are omitted.

Abbreviations: cxS1 (primary somatosensory cortex), cxS2 (secondary somatosensory cortex), cxAss (association cortex), cxCg (cingulate cortex), cxIns (insular cortex), cxM (motor cortex), inf. (inferior), lat. (lateral), med. (medial), sup. (superior).

(nWT_OVX=13, nWT=18)

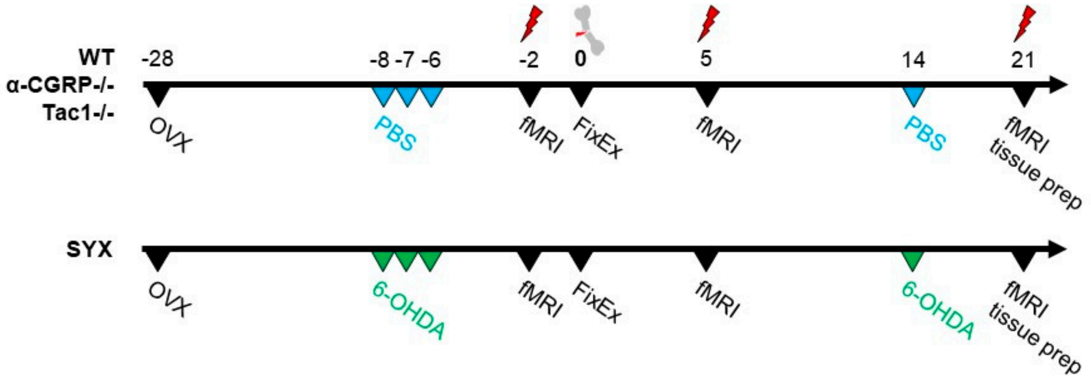

(a)

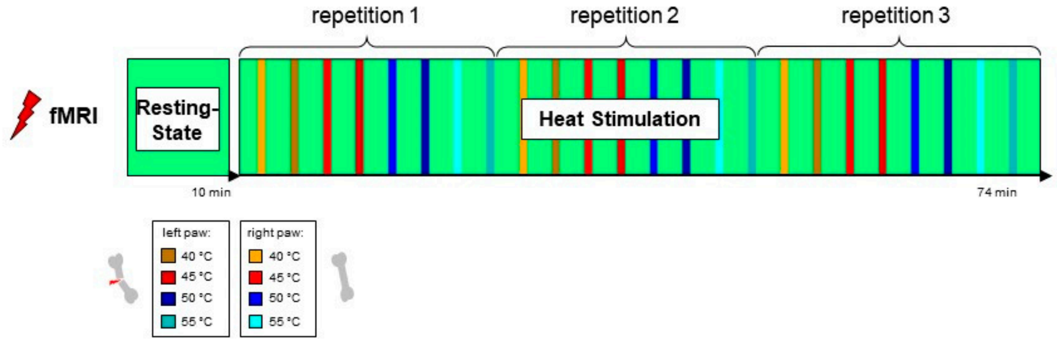

(b)

Figure S3: Experimental design. (a) Timeline: 28 days before setting stabilized fractures of the left femora (external fixator, see Surgical procedures), mice were ovariectomized (OVX). Mice of the SYX group were injected i.p. with 6-OHDA for sympathectomy, all other groups received sham injections (PBS). Two days before fracture, mice were subjected to analysis of baseline brain activity using resting-state and stimulus-driven fMRI. These measurements were repeated 5 (hyperalgesia is high) and 21 days after fracture setting (hyperalgesia is low). (b) FMRI paradigm. First, 10 min of baseline resting-state activity was acquired. During the subsequent 74 min lasting stimulus-driven fMRI measurement, mice were stimulated with three sets of four ascending temperatures, ranging from innocuous (40 and 45 °C) to noxious (50 and 55 °C) at the dorsal side of both hind paws (see FMRI preparation, protocols and stimulation paradigm).

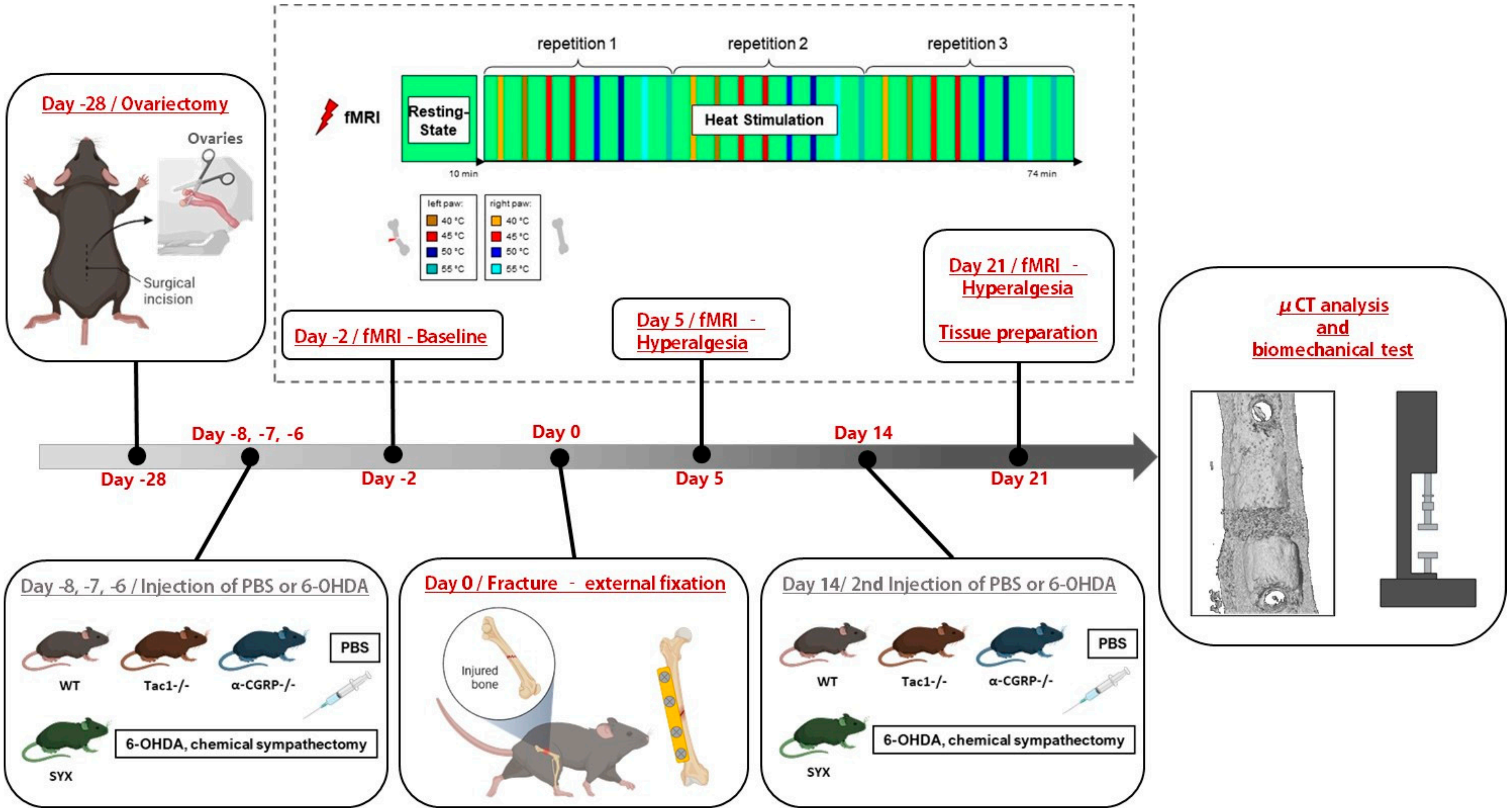

Figure S4: Schematic presentation of the experimental design. All mice (22 WT, 9 Tac1-/-,9 α-CGRP-/-; background C57Bl/6J) underwent bilateral ovariectomy 28 days before setting the fractures (= day -28). On days 8, 7 and 6 before fracture, 9 randomly chosen WT mice were injected with 80mg/kg 6-hydroxydopamine (6-OHDA; dissolved in PBS and stabilized in 0.1 % ascorbic acid) to erase sympathetic peripheral nerve endings, resulting in an 80% reduction of adrenergic neurotransmitter supply. 13 WT, 9 Tac1-/- and 9 α-CGRP-/- mice were injected with 150 µl PBS with 0.1 % ascorbic acid. On day -2, first fMRI analysis was performed for Baseline measurements (minus 1 SYX mice due to complications during the analysis). On day 0, externally stabilized femoral fractures were set in the left hind leg of all mice (external fixation MouseExFix; RISystems AG). 5 days after fracture, resting state

and hyperalgesia were analyzed using fMRI. On day 14, SYX mice received a second injection of 6-OHDA to maintain reduced adrenergic neurotransmitter supply, all other mice were injected with PBS, as described before. On day 21 resting state and hyperalgesia of 5 WT, 5 Tac1-/-, 5 α-CGRP-/- and 4 SYX mice were measured using fMRI (remaining mice were used in another study and are not counted for further analysis in this study). Afterwards mice were euthanized and both femora (left - fractured; right - non-fractured) were prepared for μCT analysis and biomechanical tests. The experimental setting allows to study the effects of sensory and sympathetic neurotransmitter on fracture healing and fracture induced hyperalgesia from day 0 to day 21 (remodeling phase). Images of mice and the biomechanical testing machine were designed using biorender.com.
